# Prevalence of Barmah Forest Virus, Chikungunya Virus and Ross River Virus Antibodies among Papua New Guinea Military Personnel before 2019 †

**DOI:** 10.3390/v15020394

**Published:** 2023-01-30

**Authors:** Joanne G. Kizu, Melissa Graham, Richard Grant, Fiona McCallum, Brady McPherson, Alyson Auliff, Peter Kaminiel, Wenjun Liu

**Affiliations:** 1 Australian Defence Force Malaria and Infectious Disease Institute, Weary Dunlop Drive, Gallipoli Barracks, Enoggera, QLD 4051, Australia; 2 Queensland Institute of Medical Research-Berghofer Medical Research Institute, Heston, QLD 4029, Australia; 3 Operational Health, Joint Health Command, Canberra, ACT 2600, Australia; 4 Health Services, Papua New Guinea Defence Force, Port Moresby 121, Papua New Guinea

**Keywords:** antibody, Barmah Forest virus, Chikungunya virus, Papua New Guinea Military Personnel, Ross River virus

## Abstract

Barmah Forest virus (BFV), Chikungunya virus (CHIKV) and Ross River virus (RRV) belong to the *Alphavirus* genus of the family *Togaviridae.* All three virus infections have been reported in Papua New Guinea (PNG) previously, but the exact prevalence and distribution of these three alphaviruses in PNG has not been established. Sera collected from 204 PNG Military Personnel (PNGMP) study participants in April 2019 was tested for the presence of anti-BFV, anti-CHIKV and anti-RRV immunoglobulin G (IgG) antibodies using commercially available enzyme-linked immunosorbent assay (ELISA) IgG detection kits, as well as for specific neutralizing antibodies (NAb) against individual viruses. Overall, sero-positivity of the sera was anti-BFV IgG 12.3% (25/204), anti-BFV NAb 8.3% (17/204); anti-CHIKV IgG 47.1% (96/204), anti-CHIKV NAb 34.8% (71/204); and anti-RRV IgG 93.1% (190/204), anti-RRV NAb 56.4% (115/204), respectively. Of the 137/204 participants that were Nab-positive for at least one virus, we identified 4 BFV, 40 CHIKV and 73 RRV single infections, and 9 RRV+CHIKV and 11 BFV+RRV double infections. The lower proportion of NAb sero-positive compared to the ELISA IgG sero-positive assay samples suggests that the currently available commercial ELISA detection kits for these three alphaviruses may not be suitable for diagnostic/surveillance purposes in endemic areas such as PNG, due to serological cross-reactivity among these three alphaviruses. Laboratory testing using known positive control sera indicated no cross-neutralization between BFV and RRV; however, some RRV or BFV single infection human sera demonstrated low-level cross-neutralization against CHIKV (the ratio of RRV/CHIKV NAb titers or BFV/CHIKV ≥ 4). Our preliminary results indicate that the majority of PNGMP have previously been exposed to RRV, with mild exposure to CHIKV and low-level exposure to BFV, suggesting that multiple alphaviruses have been circulating among PNGMP. The transmission landscapes of these three alphaviruses across PNG should be prioritized for further investigation, including identification of specific vectors and hosts that mediate human spillover in order to mitigate future outbreaks. Ongoing education regarding precautionary and protective measures are needed to better protect individuals who travel to PNG.

## 1. Introduction

Barmah Forest virus (BFV), Chikungunya virus (CHIKV) and Ross River virus (RRV) are phylogenetically and antigenically closely related mosquito-borne, single-stranded, positive-sense RNA viruses that belong to the *Alphavirus* genus of the family *Togaviridae* [1,2]. RRV and BFV, recognized as typical Australian arboviruses, are the top two causes of human arboviral diseases in Australia; ~55,000 RRV and ~15,000 BFV cases have been reported over the past decade [3]. RRV sero-prevalence has been reported outside Australia, including in French Polynesia and American Samoa [4,5] and a single BFV human case was reported in Papua New Guinea (PNG) [6]. After major outbreaks starting in 2004, CHIKV spread to subtropical areas and the western hemisphere from sub-Saharan Africa, Southeast Asia, and the Indian subcontinent and caused millions of human infections around the world. Although CHIKV is not currently endemic within Australia, imported cases from Pacific Island Countries and Territories (PICTs) into Australia have occasionally been reported [7]. Infections with these three alphaviruses cause similar clinical symptoms, such as acute febrile illness commonly associated with poly-arthralgia, fever, maculopapular rash, headache, fatigue and myalgia. These clinical symptoms are indistinguishable from flavivirus infections, such as those caused by the dengue and Zika viruses. In general, BFV infection symptoms are milder than those of CHIKV and RRV [7]. RRV and BFV disease are non-lethal and self-limiting, but CHIKV has a low rate of mortality. About ~30% of RRV and ~50% of CHIKV-infected individuals will develop chronic disease with persistent severe joint pain, tenosynovitis and incapacitating polyarthralgia that can last for months to years, resulting in a substantial impact on the quality of life of infected individuals and significant economic losses. Up to 70% of RRV and 28% of CHIKV-infected persons are asymptomatic [8,9]. The proportion of asymptomatic BFV infections has not been recorded, but is thought to be similar to RRV or higher. RRV and BFV have a natural animal–mosquito–animal transmission cycle throughout Australia [10,11], whereas CHIKV maintains a sylvatic cycle with nonhuman primates as the major reservoir host [12]. There are no specific treatments, drugs or licensed vaccines available to prevent the diseases caused by these three alphaviruses. Clinical management primarily targets the relief of symptoms. With the presence of the mosquito vector in tropical and subtropical areas around the world, these three alphaviruses will likely continue to spread to different territories from their original country of discovery and cause huge public health concerns.

As clinical symptoms of these three alphavirus infections are similar, differential diagnostic methods to identify the true virus infection are necessary. Laboratory definitive diagnosis of these alphavirus infections requires either isolation of the virus detection of viral RNA by RT-PCR testing in serum collected <6 days after onset of illness or confirmation of seroconversion (≥4-fold rise in IgG titer) against of any these viruses using paired serum samples. The detection of RRV/BFV-specific IgM and IgG antibodies is only suggestive of infection due to serological cross-reactivity among alphaviruses, with potential for associated false positive results [13]. However, the majority of clinicians in Australia still depend upon pathology laboratory detection of IgM antibodies (which normally appear in serum collected approximately >5 days after illness onset and last up to 3 months) using commercially available serological IgM test kits [14,15]. Due to the similarity of clinical symptoms, as well as overlapping mosquito vectors and geographical locations, patients presenting with the aforementioned polyarthritis symptoms are usually tested for both RRV and BFV in Australia. 

PNG is the world’s third largest island country in the Southwest Pacific, with approximately 8 million inhabitants. Arbovirus infections are a major public health concern in this country. Reports of RRV in PNG date back to the 1970–1990s, with research conducted by Australian scientists revealing that RRV is prevalent throughout the country [16,17,18]. The first outbreak of CHIKV in PNG was reported in June 2012 [19]. Since then, it spread rapidly throughout PNG. There have been occasional reports of CHIKV imported from PNG to Queensland [7]. Based on research by Indonesian scientists, CHIKV is still circulating in PNG [20]. While only a single case of BFV infection was reported from the central coast of PNG outside Australia, BFV is believed to have circulated in PNG for a long time [6,21]. 

The lack of diagnostic, clinical reporting and disease surveillance opportunities means that the distribution and prevalence of these diseases within PNG are not well known. It is important to respect the epidemic potential of these diseases and promote public awareness regarding exposure potential, so that proper measures can be implemented to prevent exposure to vectors. We conducted a population-based alphavirus sero-prevalence survey amongst PNG military personnel (PNGMP) in March-April 2019. For this study population, using known positive control sera, we measured and compared antibodies using commercial enzyme-linked immunosorbent assay (ELISA) IgG kits and neutralization assays against the alphaviruses RRV, BFV and CHIKV. 

## 2. Methods

### 2.1. Ethical Approval

The study was approved by the PNG Medical Research Advisory Committee (MRAC No. 18-21) and the Department of Australian Defence and Veteran Affairs Human Research Ethics Committee (DDVA HREC No. 084-18 and DDVA HREC 157-19). Written formal consent was obtained from all participants. 

### 2.2. Cells and Viruses

Vero cells (African green monkey kidney epithelial cells) were purchased from the American Type Culture Collection (ATCC, CCL-81) and cultured in 10% *v/v* heat-inactivated fetal calf serum (FCS, Life Technologies, Carlsbad, CA, USA)/RPMI 1640 medium (Sigma-Aldrich, St. Louis, MO, USA). A RRV QML strain (GenBank No. GQ433354), a prototype BFV strain (BH2193, GenBank No. NC_001786) (both virus strains were gifted by Professor Aaskov) and a CHIKV Reunion strain (GenBank No. DQ443544, gifted by Professor Suhrbier) were used for neutralizing antibody (NAb) assays. The virus strains were passaged three times in Vero cells and the cell culture supernatant was harvested, aliquoted and stored at −80 °C for further use. One vial of viral stock was thawed to determine virus titer by 50% tissue culture infectious dose (TCID50/mL) on Vero cells, as described previously [22]. Briefly, virus stocks were 10-fold serially diluted, and 100 µL of diluted virus was inoculated onto monolayers of Vero cells grown in 96-well plates in RPMI 1640 supplemented with L-glutamine, 5% FBS and 1% PSG, which were maintained at 37 °C, 5% CO_2_. After 4 days of incubation, cells were fixed with 3.7% formaldehyde, stained with 1% crystal violet for 1 h, washed in tap water and dried, and then infected wells were counted. The TCID50/mL were calculated according to the published Reed–Muench method [23]. 

### 2.3. ELISA

Commercially available anti-BFV and anti-RRV ELISA IgG kits from Abbott, Australia (Abbott, https://www.globalpointofcare.abbott/en/index.html) and an anti-CHIKV ELISA IgG kit from Euroimmun (Euroimmun, https://www.euroimmun.com, Lübeck, Germany) were used according to the manufacturer’s protocol for anti-RRV ELISA IgG (product code 04PE10), anti-BFV ELISA IgG (product code SD05PE10) and anti-CHIKV ELISA IgG (EI 293A-9601 G). Positivity was determined by comparing the sample result to the IgG reference sera provided (cut-off calibrators). A positive sample was defined as having a sample/calibrator absorbance ratio of ≥1.1, a negative sample was defined as having a ratio of <0.8 and an equivalent sample was defined as having a ratio of ≥0.8 to <1.1. 

### 2.4. Micro-Neutralization Assay 

Anti-BFV-, anti-CHIKV- and anti-RRV-specific NAb responses were assessed using a micro-neutralization assay, modified according to the methods described in a publication for CHIKV virus [24]. Briefly, heat-inactivated (56 °C for 30 min) serum samples were diluted 1:10 with dilution medium (DM) (RPMI supplemented with 10% fetal bovine serum, 10 mM HEPES, 20 mM L-glutamine, 100 units/mL of penicillin). Two-fold serial dilutions were performed in duplicate using 50 μL aliquots across rows A–G of the plate. Wells G1–G6 of the plate contained 50 μL aliquots of DM alone as virus controls, and wells G7–G12 contained 100 μL aliquots of DM as cell only controls. 

Volumes of 50 μL of virus stock, containing 100 TCID50 infectious doses of either BFV, CHIKV or RRV, were added to the virus control wells and to these with serially diluted sera, but not the cell-only control wells. The mixtures were incubated at 37 °C with 5% CO_2_ for 1 h to allow virus neutralization. Following neutralization, a suspension of 2 × 10^4^ Vero cells in 100 μL DM as added into each well of the 96-well plates. Ninety-six hours later, cells were fixed with 3.7% formaldehyde, stained with 1% crystal violet for 1 h, washed in tap water and dried, and then the absorbance (O.D.) of the wells was read at 595 nm using a 96-well plate spectrophotometer (Infinite M200 pro, TECAN, MA, USA). Test results were considered valid if the O.D. of virus control wells in the same plate was lower than 0.3 and the O.D. of the cell control wells was higher than 1.5. The value used to determine the presence of neutralizing antibodies was calculated according to the following formula: cut-off value = (mean O.D. of 4 virus control wells in the same plate + mean O.D. of 4 cell control wells in the same plate)/2. If the duplicate wells were both above the cut-off values, the reciprocal antibody dilution to those wells were recognized as a neutralization antibody titer for that human sample. Anti-BFV-, anti-CHIKV- or anti-RRV-specific NAb titers ≥10 were considered positive.

### 2.5. Positive and Negative Control Samples

Fourteen RRV and five BFV single infected Australian Defence Force (ADF) human serum samples were used as positive controls in this study. These serum samples were drawn from patients who had previously been diagnosed with a single virus infection by either RT-PCR or virus isolation, or who previously had a ≥ 4-fold rise in Nab titer against either RRV or BFV in our laboratory. Two CHIKV single infected serum samples obtained from a Queensland public pathology laboratory were used as CHIKV controls. Both sera were diagnosed as CHIKV infections using the Euroimmun ELISA IgM kit, and this was further confirmed by neutralization assay against BFV, CHIKV and RRV. The Panbio RRV IgG kit positive control was also included. Five ADF- ELISA IgG/IgM sera that were negative for all three alphaviruses were used as negative controls for the neutralization assay.

### 2.6. Statistical Analysis

The study population’s mean age was calculated. Anti-alphavirus NAb profiles were defined as naïve infection (no previous virus infection), single infection (infection with one virus), or multi-virus infection (>1 virus infection) based on the following criteria:

Naïve infection: NAb titers <10 for all three alphaviruses.

### 2.7. Single Virus Infection

NAb titers ≥10 to only one alphavirus or titers ≥10 to multiple viruses, but with a predominant response (≥4 times higher than the titers determined against other tested viruses between RRV and CHIKV and CHIKV and BFV, respectively). 

### 2.8. Multi-Alphavirus Infection

BFV^+^+ RRV^+^ double infection was defined as both RRV^+^ + BFV^+^ positive samples, or RRV^+^ + BFV^+^ + CHIKV^+^ samples with the RRV or BFV response being predominant over that for CHIKV.

RRV^+^ + CHIKV^+^ double infection was defined as RRV^+^ + CHIKV^+^ samples without a single predominant titer, or RRV^+^ + BFV^+^ + CHIKV^+^ samples with CHIKV being the predominant BFV. 

We analyzed the data with Graph-pad Prism version 6.03 software, using *t*-test and Chi-square tests to compare the NAb titer and NAb antibody prevalence rates, with significance set at *p* < 0.05.

## 3. Results

### 3.1. Study Population Demographics

**Figure 1:**
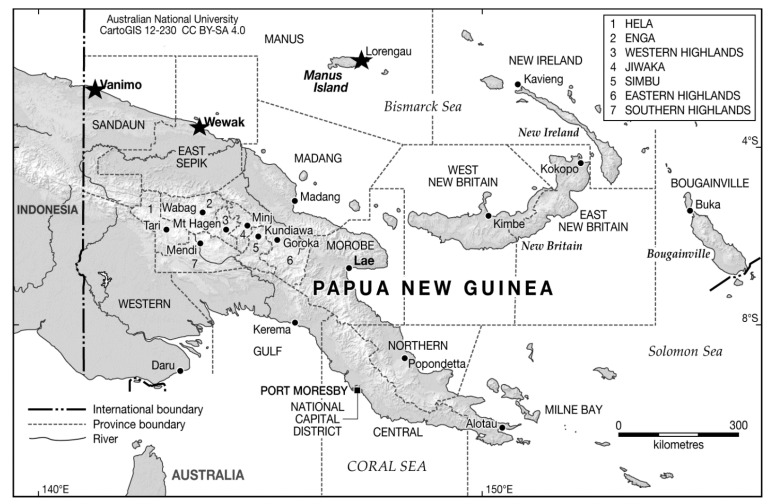
Location of serological survey of Papua New Guinea Military Personnel. An asterisk has been used to indicate the locations of Manus Island, Wewak and Vanimo.

This study was part of an infectious disease surveillance program conducted by the ADF in conjunction with the PNG Defence Force. In total, 76 PNGMP from Lombrum Naval Base, Manus Island Province, the largest of the Admiralty Islands, and 132 PNGMP from Moem Army Base (Wewak, East Sepik province), including its Vanimo outpost (Sandaun province), consented voluntarily to participate in this survey, which was conducted between March and April 2019 (Figure 1). The largest participant age group was 20–35 years old (52.9%), followed by 36-50 years old (32.8%). Participants from Manus Island were predominantly Navy (97.4%), while those from Wewak were Army (89.8%). Only one female, from Wewak, participated in the survey (Table 1). Four samples collected from PNGMP from Wewak were not analyzed for anti-RRV or anti-CHIKV NAb due to insufficient sera volume, so they were excluded. 

### 3.2. Rate of Sero-Prevalence Determined by Pathogen-Specific ELISA IgG and Pathogen-Specific Nab

**Figure 2:**
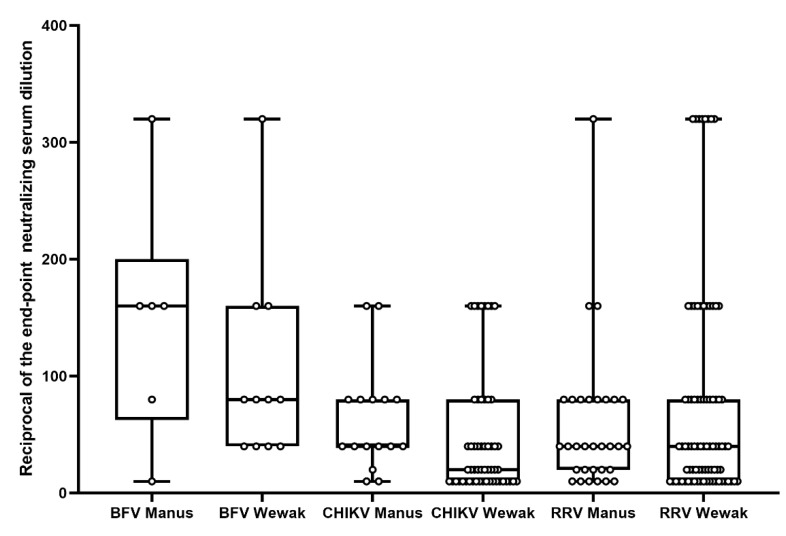
Box plot illustrates neutralizing antibody concentrations of three alphaviruses among Papua New Guinea Military Personnel located in Wewak and Manus Island, 2019. The y-axis represents the reciprocal end-point neutralizing serum dilutions. Box plots show the median (horizontal line) and the min to max of neutralizing antibody titers. All positive serum samples are included.

The prevalence rates of anti-BFV IgG and NAb against the BFV prototype strain BH2193 from 204 PNGMP samples were 12.3% (25/204) and 8.3% (17/204), respectively, with a mean NAb titer of 118 (95% CI: 71–165) (Table 2 and Figure 2). One sample tested was ELISA IgG equivalent and Nab-positive. There was no statistical difference in NAb titers (unpaired t test *p* = 0.37, *p* > 0.05) or IgG sero-positivity rates (Chi-square *X*^2^= 2.26, *p* = 0.13, *p* > 0.05) between Wewak and Manus PNGMP. We did not compare the NAb seropositivity rate between age groups due to the low sero-positivity rate. 

The prevalence rates of anti-CHIKV IgG and NAb against the CHIKV Reunion strain from 204 PNGMP samples were 47.1% (96/204) and 34.8% (71/204), respectively, with a mean NAb titer of 50 (95% CI: 38–62), (Table 2 and Figure 2). Five ELISA-negative and six ELISA-equivalent samples were Nab-positive. The prevalence of the anti-CHIKV NAb positivity rate was significantly higher in the Wewak PNGMP compared with Manus Island (*χ*^2^ = 10.1, *p* = 0.0237, *p* < 0.05). The NAb sero-positivity rate did not differ between age groups (20–35, 36–50 and 51–62 years) (Table 2 and Figure 2). 

The prevalence rates of anti-RRV IgG and NAb against a RRV QML strain from 204 PNGMP samples were 93.1% (190/204) and 56.4% (115/204), with a mean titer of 72 (95% CI: 56–88), respectively (Table 2 and Figure 2). Seventy-six samples that tested as ELISA-positive were NAb-negative. One sample was ELISA IgG-negative, but NAb-positive. The prevalence of anti-RRV NAb positivity among the participants from Manus Island and those from Wewak did not differ (Chi-square *X*^2^ = 3.4989, *p* = 0.06, *p* > 0.05). A total of 46.7% (49/105) of participants born after the largest RRV outbreak ever reported in the PICTs (1979–1981) [8] showed anti-RRV NAb, in comparison to 66.7% (66/99) of the participants born before 1982 (before the outbreak) (Chi-square *X*^2^ = 2.313, *p* = 0.1283, *p* > 0.05). The NAb titer among the participants from Wewak did not differ from that of the Manus Island participants (Figure 2, unpaired t-test *p* = 0.230, *p* > 0.05). 

As only one female participant was enrolled, analysis based on sex was not performed. 

### 3.3. Cross-Reactivity of NAbs and Double Infections

None of our fifteen RRV IgG-positive control samples cross-neutralized BFV, but eight of them cross-neutralized CHIKV, showing low NAb titers ≤10 and a RRV/CHIKV titer ratio of ≥4-fold. None of our six BFV single infection IgG-positive samples neutralized RRV, but one sample cross-neutralized CHIKV, showing a low NAb titer (≤10) and a BFV/CHIKV titer ratio of ≥4-fold. Our two CHIKV single infection serum controls cross-neutralized RRV, showing NAb titers of ≤20 and a CHIKV/RRV NAb titer ration of ≥4 fold, but they did not neutralize the BFV prototype strain. Five ADF sera controls collected before personnel deployment to PNG in 2019 did not neutralize any virus (Table 3).

Based on our positive control results, we defined naïve, single or double infections as stated in the methods section. Sixty-seven samples were determined to be infection-naïve. Of the 137 subjects that were NAb-positive for at least one virus, we identified four BFV, 40 CHIKV and 73 RRV single infections, and 9 RRV+CHIKV and 11 BFV+RRV double infections (Table 3 and Table 4). 

Although three samples (sample numbers 105, 170 and 205) from Wewak were triple NAb-positive; the titers for RRV (sample number 105 and 205) or BFV NAb (sample number 170) were ≥ 80 and ≥ 4-fold the CHIKV NAb titer. Therefore, all three samples were recognized as double RRV-BFV infections (Table 4). 

## 4. Discussion

Our alphavirus sero-prevalence study identified serological evidence for the circulation all three alphaviruses, BFV, CHIKV and RRV, among PNGMP before 2019. The variable exposure rates of PNGMP to these three alphaviruses indicate that PNG is a hyper-endemic country for these alphaviruses. This study generated alphavirus sero-prevalence data when little or no data were previously available.

The proportion of samples showing anti-BFV, anti-CHIKV and anti-RRV ELISA IgG positivity was higher than the proportion exhibiting NAb positivity. This is likely because these three alphaviruses are antigenically closely related to each other, and many commercial diagnostic tests kits show serological cross-reactivity among alphaviruses [25]. In Australia, most BFV and RRV IgG responses are determined using indirect ELISA kits manufactured by PanBio [11]. The company claims that the sensitivities (true positive rates) of both their anti-BFV and anti-RRV IgG ELISA kits are 100% and that the specificities (true negative rates) are 100% and 93%, respectively. However, 100% reliability in determining participant BFV compared with RRV exposure history using ELISA testing is currently not achievable due to the possibility of false-positive results. Cross-reactivity between RRV and BFV ELISA testing has also been reported [13,15,26]. For example, a high BFV IgM false positive rate of 19% (7/37) was reported by the Western Australian Health Department despite PanBio claims that the anti-BFV IgM kit has 95% specificity [27]. A lower sensitivity of 85% for PanBio ELISA RRV IgG was reported following testing of samples collected in 1996 and 1997, using an HI test as the comparator [8]. One paper reported that 93% of anti-RRV IgG positive and 28% of anti-BFV IgG human samples reacted positively on CHIKV-infected cells by immunofluorescence assay [28]. The Euroimmun’s anti-CHIKV IgG instruction book indicated that 30% of RRV, 50% of Mayaro virus and 2.2% of BFV human samples were shown to be anti-CHIKV IgG-positive, but claimed sensitivity and specificity values for their anti-CHIKV IgG kit as 95.8–98.6% and 98–99.8%, respectively [29]. We found that amongst fifteen RRV-positive controls, six were also ELISA-positive for anti-CHIKV IgG and two were anti-BFV IgG-positive. Two CHIKV-positive controls also tested anti-RRV IgG positive by ELISA (Table 3). Our results imply that the current commercially available ELISA IgG detection kits for BFV, CHIKV and RRV may not be suitable for diagnostic purposes in countries such as PNG where these three alphavirus are endemic, possibly due to serological cross-reactivity among alphaviruses. Ideally, any ELISA IgG positive alphavirus samples should be confirmed serologically by NAb assay. Our finding of positive neutralizing results for one BFV, eleven CHIKV and one RRV ELISA-negative or -equivalent samples could be due to antigenic differences between the antigens used for ELISA assays, the circulating virus linages in PNG or the laboratory isolates used in the NAb assays [30]. In addition, known difficulties with alphavirus ELISA assay sensitivity may have contributed to this result, with stated sensitivity values of only 88% for the anti-CHIKV IgG Euroimmun ELISA [31] and 85% for the anti-RRV IgG PanBio ELISA [8]. 

Anti-viral NAb assay remains the gold standard test method for confirmation of recent/past alphavirus infection, with high specificity and correlation with immune protection. This test type allows for the determination of antibody titer levels, although it cannot differentiate between antibody subclass types, e.g., IgM and IgG. Cross-protective NAb between CHIKV and RRV has been previously reported. Anti-CHIKV monoclonal antibody (CHK-265) can neutralize RRV infections in cell culture and is able to protect against disease caused by RRV in mice [32]. Pre-existing immunity to RRV infection reduces clinical disease following challenge with CHIKV [33]. However, NAb cross-reactivity among the antigenically closely related CHIKV, RRV and BFV has not been studied extensively, partially due to the lack of CHIKV transmission in Australia. Our results indicate no cross-neutralization using single anti-RRV or anti-BFV human sera, but that some anti-RRV or anti-BFV NAb sera can cross-neutralize CHIKV (Table 3). A comparison of NAb cross-reactivities showed NAb RRV infection sera with RRV/CHIKV endpoint titer ratios of ≥4-fold difference, BFV infection sera with BFV/CHIKV titers of ≥4 fold differences and CHIKV infection samples with CHIKV/RRV titers of ≥4 fold differences. Therefore, we applied the traditional ≥4 fold difference rule regarding NAb titers against the real infecting virus compared with titers achieved against the possible cross-reactive virus to determine the single infections in the PNGMP samples (Table 4). 

Co-circulation and simultaneous co-infection of the dengue, chikungunya and zika viruses in patients with febrile syndrome has been reported [34,35]. Double or triple alphavirus infections with one alphavirus at an earlier time and late infection with another alphavirus are possible due to the sharing/overlapping mosquito vectors and geographical locations, particularly in endemic areas such as PNG. In these scenarios, double/triple-positive NAb results might not be caused by cross-reactivity of the corresponding antibodies. Double BFV, CHIKV or RRV infections have not been reported, partially due to the fact that CHIKV is not endemic in Australia. We identified 11 PNGMP samples that had RRV+CHIKV double infection, and nine samples that were RRV+BFV exposures, with no sample determined to have triple infection. 

The BFV sero-prevalence determined following testing of healthy blood donors from Queensland (the area in Australia with the highest BFV endemicity) using the PanBio anti-BFV IgG kit in) was IgG 1.7% [15], and no BFV sero-survey has previously been conducted outside Australia. Our survey result of 8.3% NAb sero-prevalence (probably higher as we only tested anti-BFV NAb of ELISA IgG^+^ and equivalent samples) suggests that BFV has been circulating among PNGMP. 

RRV sero-surveys suggest that anti-RRV NAbs were present in the general population of many regions of PNG sampled from 1960-1969 [36,37]. A later study found a 59% RRV sero-prevalence in the Southern Highlands of PNG in 1991 [36]. The first and only isolation of RRV from PNG to date was in 1997, from a pool of *Anopheles farauti* mosquitoes collected at Wandoo village in the Bensbach region of the Western Province [38]. Since then, there have been no documented serosurveys conducted in PNG. The PNGMP anti-RRV NAb prevalence (56.4%) (Table 2) is much higher than the reported 8.4% RRV IgG sero-prevalence in healthy blood donors in Queensland (the area in Australia with the highest RRV endemicity, detected using the PanBio anti-RRV IgG ELISA kit) [15], suggesting that RRV has been circulating among PNGMP for a long time. 

Previous CHIKV sero-survey results conducted in countries on different continents reported sero-prevalence rates ranging from 10.2% to 75%, depending on the subpopulation studied, the timing of the study and the intensity of virus circulation [39]. These studies applied either indirect immunofluorescence IgG/IgM or Euroimmun IgG/IgM ELISA kits for detection. Nevertheless, the 34.8% anti-CHIKV NAb prevalence amongst PNGMP implies that CHIKV has been circulating since its first reported outbreak in 2012 [19].

The finding of higher CHIKV sero-prevalence in Wewak PNGMP may suggest an increased transmission risk on the mainland of PNG rather than on the remote Manus Island. We do not know when and where exposure occurred, as the deployment service history of individuals was not obtained. The higher exposure could also be due to age differences, as the participants from Manus Island were younger than those from Wewak (Table 1). Increased international travel; density of population, which increases the possibility of human–mosquito–human transmission; and other related environmental factors, such as a possible predominance of CHIKV vector mosquito species *Aedes aegypti* and *Ae. albopictus* on the main island, may also have contributed to differing exposure levels. 

With the recent joint commitment between the USA, Australia and PNG to develop a naval base on PNG’s Manus Island, there is an ongoing risk to military personnel without natural immunity through prior exposure traveling to Manus. There is also a potential risk for RRV and BFV to be exported beyond the Pacific region through infected, asymptomatic military personnel returning to countries where competent vector mosquitoes have been found [40,41]. There is emerging evidence that RRV can be transmitted in areas where the natural marsupial hosts are absent [10]. The largest outbreak of RRV infection ever recorded occurred in the Pacific during 1979–1981 [42], and this provides a distinct warning of the epidemic potential of these alphaviruses. The recent experience with Zika and CHIKV underscores the serious threat posed to global health by the potential for previously obscure arboviruses, such as RRV and BFV, to move from their historical transmission locations into other parts of the world [43,44]. 

These preliminary findings require further support from additional investigations, as the present study had a small sample size and participants were limited to PNGMP. However, the results may provide a representative view of the local PNG population, as PNGMP are recruited from the general population. The authors intend to expand their arbovirus surveillance program in PNG to include identification of antibody prevalence, arbovirus transmission, dominant circulating strains and mosquito population behaviors in PNGMP from other locations. 

## Figures and Tables

**Table 1:** Demographics of the study population.

Blood Donors	Manus Island Barracks	Wewak Barracks	Total Number
Number of blood donors	76	128	204
Percentage %	36.4% (76/204)	63.6% (128/204)	100%
Sex			
Male	100% (76/76)	99% (127/128)	99.5% (203/204)
Female	0% (0/76)	0.8% (1/128)	0.5% (1/204)
Age			
Range	20–61	21–59	21–61
Mean	35.2	39.2	37.5
Median	29	41.5	34
Age group			
20–35	65.8% (50/76)	45.3% (58/128)	52.9% (108/204)
36–50	18.4% (14/76)	41.4% (53/128)	32.8% (67/204)
51–61	15.8% (12/76)	13.3% (17/128)	14.2% (29/204)
Unit			
Navy	97.4% (74/76)	0% (0/128)	36.2% (74/204)
Army (2RPIR)	1.3% (1/76)	89.8% (115/128)	56.9% (116/204)
DSS	0% (0/76)	9.4% (12/128)	5.9% (12/204)
Other	1.3% (1/76)	0.8% (1/128)	1% (2/204)

RPIR: Royal Pacific Islands Regimen; DSS: Defence Special Service.

**Table 2:** Prevalence of anti-Barmah Forest virus, anti-Chikungunya virus and anti-Ross River virus antibodies, observed in 204 Papua New Guinea military participants in this study in 2019.

Military Participants	Manus Island	Wewak	Total
Anti-Barmah Forest virus			
ELISA IgG+	17.1% (13/76)	9.4% (12/128)	12.3% (25/204)
ELISA IgG±	2.6% (2/76)	0.8% (1/128)	1.5% (3/204)
ELISA−	80.2% (61/76)	89.8% (115/128)	86.3% (176/204)
Neutralizing+ **	40% (6/15)	84.6% (11/13)	60.7% (17/28)
ELISA+, neutralizing+	38.5% (5/13)	91.7% (11/12)	64% (16/25)
ELISA±, neutralizing+	50% (1/2)	0% (0/1)	33.3% (1/3)
ELISA−, neutralizing+	N.D.	N.D.	N.D.
Suggestive neutralizing+/group	7.9% (6/76)	8.6% (11/128)	8.3% (17/204)
Anti-Chikungunya virus			
ELISA IgG+	39.5% (30/76)	51.6% (66/128)	47.1% (96/204)
ELISA IgG±	13.1% (10/76)	10.2% (13/128)	11.2% (23/204)
ELISA−	47.4% (36/76)	38.3% (49/128)	41.7% (85/204)
Neutralizing+ *	21.1% (16/76) *	43% (55/128) *	34.8% (71/204)
Neutralizing−	78.9% (60/76)	57% (73/128)	65.2% (133/204)
ELISA+, neutralizing+	18.4% (14/76)	35.9% (46/128)	29.4% (60/204)
ELISA±, neutralizing+	0% (0/76)	4.7% (6/128)	2.9% (6/204)
ELISA−, neutralizing+	2.6% (2/76)	2.3% (3/128)	2.5% (5/204)
Group of anti-CHIKV Nab-positive			
Age 20–35 group	26% (13/50)	50% (29/58)	38.9% (42/108)
Age 36–50 group	7.1% (1/14)	34% (18/53)	28.4% (19/67)
Age 51–62 group	16.7% (2/12)	47.1% (8/17/)	34.5% (10/29)
Anti-Ross River virus			
ELISA IgG+	89.5% (68/76)	95.3% (122/128)	93.2% (190/204)
ELISA IgG±	3.9% (3/76)	1.6% (2/128)	2.5% (5/204)
ELISA−	6.6% (5/76)	3.1% (4/128)	4.4% (9/204)
Neutralizing+	40.8% (31/76)	65.6% (84/128)	56.4% (115/204)
Neutralizing−	59.2% (45/76)	34.4% (44/128)	43.6% (89/204)
ELISA+, neutralizing+	40.8% (31/76)	64.8% (83/128)	55.9% (114/204)
ELISA+, neutralizing−	48.7% (37/76)	30.5% (39/128)	37.3% (76/204)
ELISA±, neutralizing+	0% (0/76)	0% (0/128)	0% (0/204)
ELISA−, neutralizing+	0% (0/76)	0.8% (1/128)	0.5% (1/204)
Nab of group born before 1982	40.7% (11/27)	76.4% (55/72)	66.7% (66/99)
Nab of group born after 1982	40.8% (20/49)	51.7% (29/56)	46.7% (49/105)

* Statistically significant difference between participants from Manus Island and Wewak Barracks. ** Only the ELISA-positive and -equivalent samples were tested with a neutralization assay for Barmah Forest virus.

**Table 3:** Anti-Barmah Forest virus, anti-Chikungunya virus and anti-Ross River virus antibody observations for our RRV-, BFV- and CHIKV-infected personnel, as well as negative controls.

Serum No.	Anti-BFV		Anti-RRV		Anti-CHIKV	
	IgG	Nab	IgG	Nab	IgG	Nab
ADF-RRV1	−	0	+	320	+	0
ADF-RRV2	−	0	+	320	+	0
ADF-RRV3	−	0	+	320	−	0
ADF-RRV4	−	0	+	320	+	0
ADF-RRV5	−	0	+	160	−	0
ADF-RRV6	−	0	+	640	±	0
ADF-RRV19-1	−	0	+	160	+	10
ADF-RRV19-3 Acute phase #	−	0	−	10	−	10
ADF-RRV19-3 convalesce	+	0	+	320	−	10
ADF-RRV19-6	+	0	+	320	−	10
ADF-RRV19-7 Acute phase #	−	0	+	20	−	10
ADF-RRV19-7 covalence	−	0	+	80	−	0
ADF-RRV19-8 acute phase #	−	0	−	10	−	10
ADF-RRV/19-8 convalesce	−	0	+	640	+	10
Panbio RRV Kit positive control	−	0	+	320	+	10
ADF-BFV1	+	160	−	0	−	0
ADF-BFV2	+	80	±	0	±	0
ADF-BFV3	+	40	−	0	±	0
ADF-BFV4	+	160	−	0	−	0
ADF-BFV5	+	320	−	0	−	0
ADF-BFV19-9	+	160	−	0	−	10
CHIKV-path1	−	0	+	10	+	640
CHIKV-path2	±	0	+	20	+	640
Negative 1	−	0	−	0	−	0
Negative 2	−	0	−	0	−	0
Negative 3	−	0	−	0	−	0
Negative 4	−	0	−	0	−	0
Negative 5	−	0	−	0	−	0

+ = positive; − = negative; ± = equivalent; # both anti-RRV and anti-CHIKV IgM-positive. ADF: Australian Defence Force; path: Queensland public pathology laboratory.

**Table 4:** Co-infection of Barmah Forest virus, Chikungunya virus and Ross River virus among Papua New Guinea military personnel in 2019.

Military Participants	Manus	Wewak	Total
Single BFV infection	2.6% (2/76)	1.6% (2/128)	2% (4/204)
Single CHIKV infection	19.7% (15/76)	19.5% (25/128)	19.6% (40/204)
Single RRV infection	30.3% (23/76)	39.1% (50/128)	35.8% (73/204)
BFV + CHIKV double infection	0% (0/76)	0% (0/128)	0% (0/204)
BFV + RRV double infection	2.6% (2/76)	5.5% (7/128)	4.4% (9/204)
CHIKV + RRV double infection	0% (0/76)	8.6% (11/128)	5.4% (11/204)
BFV + CHIKV + RRV triple infection	0% (0/76)	0% (0/128)	0% (0/204)

## Data Availability

The data are included within the manuscript.
